# Host genotype interacts with aerial spore communities and influences the needle mycobiome of Norway spruce

**DOI:** 10.1111/1462-2920.15974

**Published:** 2022-03-22

**Authors:** Miguel A. Redondo, Jonàs Oliva, Malin Elfstrand, Johanna Boberg, Hernán D. Capador‐Barreto, Bo Karlsson, Anna Berlin

**Affiliations:** ^1^ Department of Forest Mycology and Plant Pathology Swedish University of Agricultural Sciences Uppsala Box 7026, 750 07 Sweden; ^2^ Department of Crop and Forest Sciences University of Lleida, Alcalde Rovira Roure 191 Lleida 25198 Spain; ^3^ Joint Research Unit CTFC‐AGROTECNIO Alcalde Rovira Roure 191, Lleida 25198 Spain; ^4^ Skogforsk Svalöv Ekebo 2250, 268 90 Sweden

## Abstract

The factors shaping the composition of the tree mycobiome are still under investigation. We tested the effects of host genotype, site, host phenotypic traits, and air fungal spore communities on the assembly of the fungi inhabiting Norway spruce needles. We used Norway spruce clones and spore traps within the collection sites and characterized both needle and air mycobiome communities by high‐throughput sequencing of the ITS2 region. The composition of the needle mycobiome differed between Norway spruce clones, and clones with high genetic similarity had a more similar mycobiome. The needle mycobiome also varied across sites and was associated with the composition of the local air mycobiome and climate. Phenotypic traits such as diameter at breast height or crown health influenced the needle mycobiome to a lesser extent than host genotype and air mycobiome. Altogether, our results suggest that the needle mycobiome is mainly driven by the host genotype in combination with the composition of the local air spore communities. Our work highlights the role of host intraspecific variation in shaping the mycobiome of trees and provides new insights on the ecological processes structuring fungal communities inhabiting woody plants.

## Introduction

Plant associated microorganisms influence host fitness (Bulgarelli *et al*., 2013; Vandenkoornhuyse *et al*., 2015; Bacon and White, 2016; Trivedi *et al*., 2020). Among the microorganisms living inside of plants, leaf‐inhabiting fungi comprise a set of endophytic‐pathogenic‐saprotrophic species of which some can provide the plant with stress tolerance, growth enhancement or pathogen protection (Arnold *et al*., 2003; Koskella *et al*., 2017; Oliva et al., 2020b). The composition of the leaf mycobiome is primarily driven by the host species (Christian *et al*., 2016; David *et al*., 2016; U'Ren *et al*., 2019), in some cases following a phylogenetic signal across different plant species (Liu *et al*., 2019). However, the drivers that shape the tree microbiome within a host species are under investigation. Several studies have shown that the mycobiome composition is determined by the host genotype (Cordier *et al*., 2012; Bálint *et al*., 2013; Rajala *et al*., 2013; Horton *et al*., 2014; Sapkota *et al*., 2015; Cregger *et al*., 2018). Other studies have highlighted the role of the physiological status of the tree or environmental conditions determining the composition of the plant mycobiome (Rajala *et al*., 2014; Eusemann *et al*., 2016; Kovalchuk *et al*., 2018; Barge *et al*., 2019; Würth *et al*., 2019; Oono *et al*., 2020). In reality, the phyllosphere mycobiome is probably the result of the joined effects of both host genetic variation and environmental factors (Ahlholm *et al*., 2002; Rajala *et al*., 2013; Bálint *et al*., 2015; Wagner *et al*., 2016; Hamonts *et al*., 2018).

A combined effect of the host genetic variation and environmental factors on the assembly of the leaf mycobiome can be the result of at least two non‐exclusive mechanisms (Wagner *et al*., 2016). On the one hand, it could be that environmental factors affect the composition of ambient fungal communities across sites. As a result, genotypes recruit fungi from the taxa available at a particular site, and therefore the differences in the composition of leaf mycobiome between genotypes are site dependent, i.e., in one site two genotypes may have a very similar microbiome, while in another site, the same genotypes will have very different mycobiome. On the other hand, it could be that the environment does not alter the composition of ambient fungal communities, but it affects the expression of plant phenotypic traits that are important for the recruitment of certain fungal taxa (Unterseher *et al*., 2016; Li *et al*., 2018). As a result, a genotype that develops a similar phenotype in different sites will tend to have a more similar community than a genotype that develop different phenotypes. The degree to which either of these mechanisms contributes to the assembly of the leaf mycobiome on trees remains unclear.

Under natural conditions, it is likely that trees are exposed to different air mycobiome across sites depending on the surrounding vegetation (Redondo *et al*. 2020). These differences in the air mycobiome will affect the composition of the tree mycobiome depending on the strength of the genetic control. If the effect of the host genotype is low, all tree genotypes at a site will have similar mycobiome, and if the air mycobiome is different across sites, the needle mycobiome of the tree genotypes would be different between sites. In contrast, if the genetic control on the microbiome is substantial, the overall effect of the local air mycobiome and interaction between genotype and air mycobiome would be less important. A strong genotypic effect could overrule any site effect, so that trees growing at distant sites end up with a very similar mycobiome. Finally, it could be that the air mycobiome substantially differ across sites and even under a strong tree genetic control, the needle mycobiome of a certain host genotype cannot become similar at different sites.

The aim of this study was to understand the ecological mechanisms contributing to the assembly of the tree mycobiome. For this, we determined the relative effect of host genotype, site, host phenotypic traits, and composition of the air mycobiome on the assembly of the needle mycobiome of Norway spruce trees (*Picea abies* (L) Karst.). We hypothesized that (i) the needle mycobiome is genetically determined and that (ii) the effect of the host genotype on the needle mycobiome depends on the composition of the local air mycobiome.

**Fig. 1:**
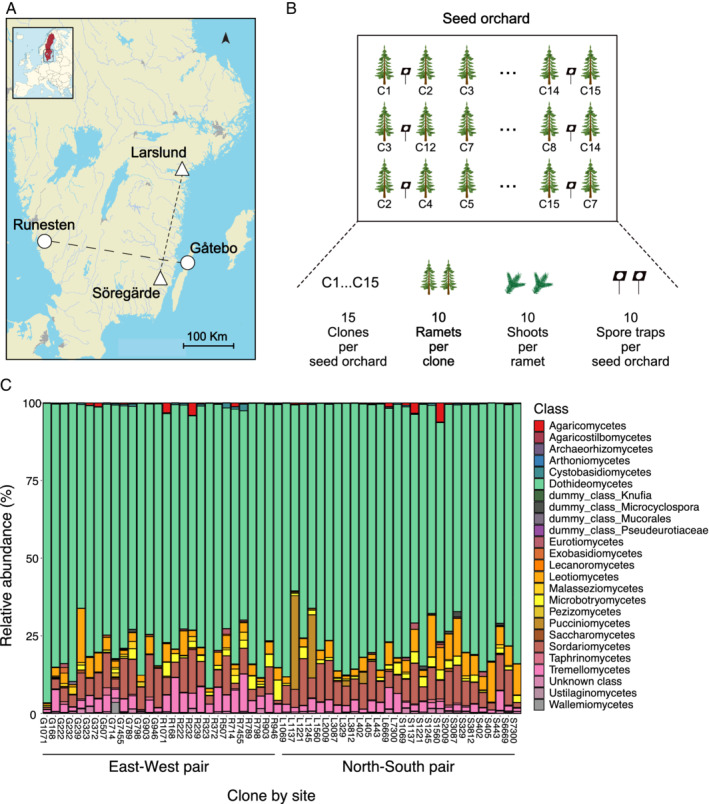
Experimental design and sequencing output. A. Distribution of the four seed orchards (sites) across Southern Sweden. Pairs of sites depicted with the same shape (triangle or circle) contain the same sampled Norway spruce clones. Analyses were done separately for each pair of sites. B. Illustration of the sampling scheme within each site. Fifteen Norway spruce genotypes (clones) were selected and 10 vegetatively propagated biological replicates (ramets) from each clone were sampled, i.e. a total of 600 trees. From each ramet, 10 current year shoots were collected, and the needles were pooled prior to DNA extraction. The final dataset included 421 samples (Supplementary Table S1). In addition, 10 passive spore traps were placed evenly across each site and collected weekly from May to July to capture the composition of the deposited fungal spores. C. Barplot of the relative abundance of fungal classes in the needle mycobiome of each clone for each of the sites. Labels in *x*‐axis display each of the clones by site. G = Gåtebo, R = Runesten, L = Larslund, S = Söregärde. Dummy class refer to the dummy nodes that Protax creates to fill gaps in taxonomy annotations (Abarenkov *et al*., 2018). The exact proportions of each fungal class can be found in Supplementary Table S2. Although we display the fungal OTUs grouped by classes, all analyses of this study were done at OTU level.

To test these hypotheses, we utilized four seed orchards containing vegetatively propagated genotypes (hereinafter ‘clones’) that belong to the Southern Swedish Norway spruce breeding programme (Chen *et al*., 2019, Rosvall, 2019). The clones have previously been characterized both genetically and phenotypically (Chen *et al*., 2019; Milesi *et al*., 2019). The establishment of the sites was done in a pairwise design (Fig. 1) and we sampled 10 trees (ramets) from each of the 15 Norway spruce clones shared between the two sites in the North–South pair (Larslund and Söregärde), and additional 15 clones shared between the East–West pair of sites (Gåtebo and Runesten). We used metabarcoding to characterize both the needle mycobiome of the clones and the air mycobiome in the Norway spruce seed orchards. Since the same clones of Norway spruce were replicated across two sites, we could disentangle the effect of the host genotype from that of the site, and phenotypic traits such as tree size and crown health. Using weekly spore trap collections during the shoot elongation period, we characterized the temporal variation of the air mycobiome being deposited at each site and analysed their contribution to the assembly of the needle mycobiome and their interplay with the different clones. As the Norway spruce clones were randomly distributed within each of the sites, we could test whether the relative location of a tree within the site determined the composition of the needle mycobiome to a greater degree than the tree genotype or phenotypic traits. Finally, by annotating the OTUs into guilds, we could test the relative importance of clone, site and their interaction on the assembly of relevant functional guilds.

## Results

### Drivers of community composition of needle mycobiome across sites

**Fig. 2:**
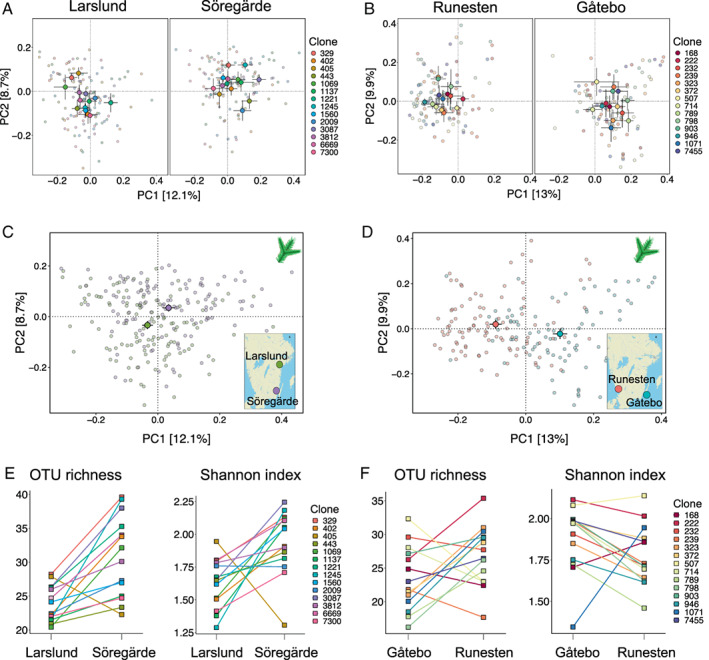
Alpha and beta diversity comparisons between clones across sites for the north–south (A,C,E) and the east–west (B,D,F) pairs of sites; (A–D) are ordination plots of a principal coordinate analysis of the needle mycobiome separated clone by site (A and B) and site (C and D). The main circles and bars represent the centroid and standard error of the samples for each of the sites or clones. The small circles represent each of the samples; (E and F) show differences in OTU richness and Shannon index for each clone on each site. The squares represent the mean values of OTU richness or Shannon index. The squares are connected by the same colour line to display differences in mean values across sites.

**Fig. 3:**
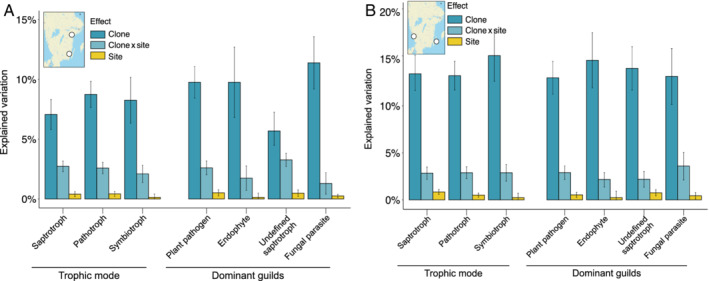
Variation explained by clone, site and their interaction on the OTUs grouped by trophic mode and guilds for the (A) North–South and the (B) East–West pair of sites. Error bars indicate standard error.

We studied the composition of the needle mycobiome of Norway spruce utilizing clones in the Norway spruce breeding programme in Sweden (Chen *et al*., 2019). We sampled and sequenced individually the needle mycobiome from each biological replicate (ramet) of each of the Norway spruce clones per site, i.e., a total of 600 samples (Fig. 1). We were able to obtain and analyse the needle mycobiome from at least three ramets per clone for 14 clones at each site pair, resulting in a total of 421 samples (Supplementary Table S1). Here, the term needle mycobiome refers to the different fungal species present inside the needles. We found that the composition of the needle mycobiome was mainly determined by host genotype, i.e., the particular Norway spruce clone (Table 1, Fig. 2A and B; Supplementary Fig. S2). The variation explained by the clone was larger than the one of the site and their interaction across trophic modes and dominant fungal guilds (Fig. 3). In terms of alpha diversity, no consistent effects of the clone and measured phenotypic traits were found across pairs of sites (Table 1, Fig. 2E and F). Across all clones, the needle mycobiome was dominated by fungal species belonging to the Dothideomycetes class, followed by Sordariomycetes, Leotiomycetes and Tremmellomycetes (Fig. 1C; Supplementary Table S2). The most dominant taxa belonged to the genus *Cladosporium*, *Aureobasidium*, *Celosporium* and *Sydowia* (Supplementary Table S3). Regarding the proportions of fungal trophic modes and dominant guilds, taxa that had a pathotrophic, saprotrophic and symbiotrophic lifestyle represented 80%, 69% and 57.5% of the total reads, respectively. Plant pathogens, endophytes, undefined saprotrophs and fungal parasites guilds accounted for 72.9%, 54.7%, 17.5% and 4.6% of total reads, respectively (the sum of the percentages exceeds 100% because some OTUs belonged to more than one trophic mode and/or guild).

**Table 1:** Summary of the ANOVA and PERMANOVA table for alpha and beta diversity, respectively.

	Alpha diversity																Beta diversity						
	North–South sites								East–West sites								North–South sites				East–West sites		
	OTU richness				Shannon index				OTU richness				Shannon index										
	df	*F*	*P*		df	*F*	*P*		df	*F*	*P*		df	*F*	*P*		df	*R* ^2^	*P*		df	*R* ^2^	*P*
Read count	1	85.6	**<0.001**		1	3.12	0.079		1	42.7	**<0.001**		1	5.79	**0.018**		1	0.021	**0.001**		1	0.028	**0.001**
Clone	13	0.88	0.58		13	1.54	0.11		13	1.39	0.17		13	1.08	0.37		13	0.082	**0.001**		13	0.083	**0.004**
Site	1	1.86	0.17		1	3.01	0.084		1	1.22	0.27		1	0.28	0.59		1	0.05	**0.001**		1	0.06	**0.001**
Diameter at breast height (dbh)	1	5.49	**0.02**		1	2.6	0.11		1	0.42	0.52		1	0.76	0.39		1	0.0056	0.064		1	0.0036	0.78
Crown health	1	1.63	0.2		1	1.39	0.24		1	0.48	0.49		1	0.66	0.42		1	0.0043	0.27		1	0.0054	0.3
Clone × site	13	1.69	0.065		13	2.86	**<0.001**		13	1.09	0.38		13	0.91	0.55		13	0.068	**0.001**		13	0.074	**0.022**
Residuals	206	‐	‐		206	‐	‐		153	‐	‐		153	‐	‐		206	0.77	‐		153	0.74	‐

Analyses were performed on each pair of sites separately. Bold letters represent significant differences at *P* < 0.05.

**Fig. 4:**
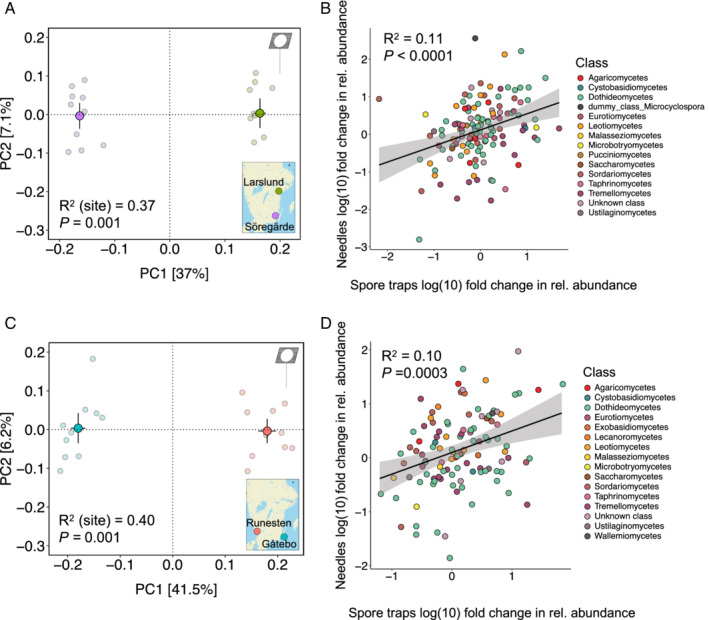
(A and C) Comparison of spore communities between sites and (B and D) association between spore communities and needle communities across sites; (A and C) are ordination plots of a principal coordinate analysis for each pair of sites. The main circles and bars represent the centroid and standard error of the samples from each site. The small circles represent each of the samples. The *R* ^2^ and *P* values are based on a PERMANOVA analysis performed with the adonis2 function in R; (B and D) are correlations of fold changes in spore and needle communities across sites for the (B) north–south and (D) east–west pair of sites. Each circle represents an OTU that is coloured based on fungal classes. Dummy class refer to the dummy nodes that Protax creates to fill gaps in taxonomy annotations (Abarenkov *et al*., 2018).

We characterized the air mycobiome during the bud flushing and shoot elongation period in all four sites (May to July) and tested its association with the needle mycobiome. The composition of both the needle (Table 1, Fig. 2C and D) and air mycobiome (Fig. 4A and C; Supplementary Fig. S3) differed across sites. Overall, air and needle mycobiome shared ca. 80% of the OTUs (527 shared OTUs and 91 and 50 unique for needle and spore traps, respectively). In fact, the needle mycobiome was associated with the composition of the local air mycobiome across all sites (Supplementary Table S4). In each pair of sites, we found that the relative abundance of the OTUs in the needle mycobiome was associated with the relative abundance in the air communities (Fig. 4B and D). We further analysed the similarities between the needle mycobiome sampled in August with the air mycobiome for each month between May and July. The needle mycobiome in August tended to be more similar to the air mycobiome from May and June than from July (Supplementary Fig. S4). Besides the effect of the air mycobiome on the phyllosphere mycobiome, we found that climatic and weather variables also had an influence. In general, a stronger influence of climate than weather was found (*R* ^2^ of 0.088 vs 0.0073 for climate and weather, respectively, Supplementary Table S4).

**Fig. 5:**
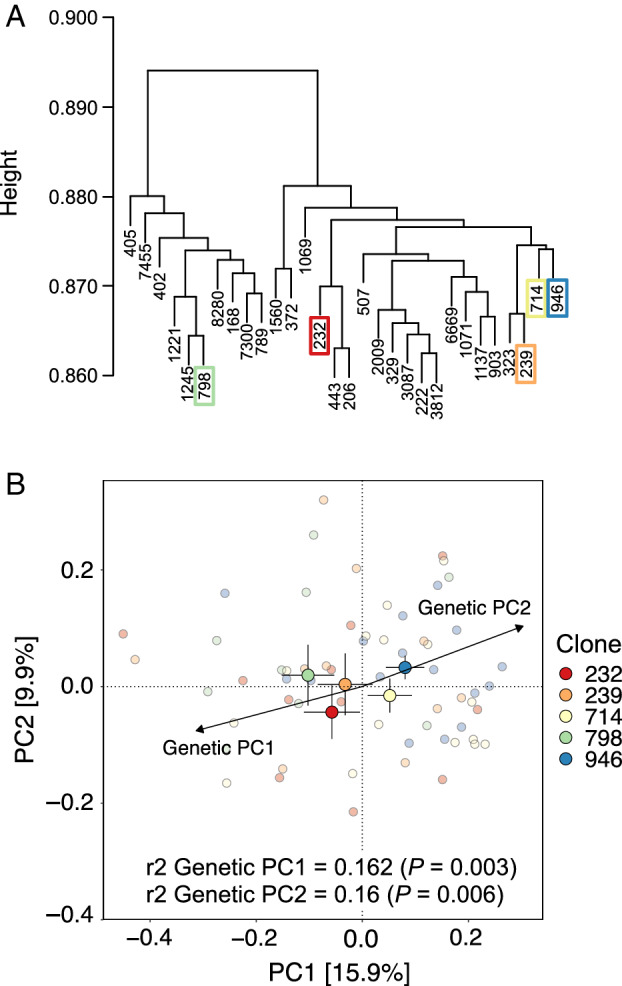
Association between genetic similarity and mycobiome composition for the five clones that had similar diameter at breast height between the two sites in the East–West pair. A. Dendrogram of the hierarchical clustering of all clones of the study based on the IBS distance matrix. The five clones selected for analyses are marked with coloured square. B. Ordination plot based on a principal coordinate analysis of the needle mycobiome. The main circles and bars represent the centroid and standard error of the samples for each of the clones averaged by site. The small circles represent each of the samples. Also shown are the correlations (*r* ^2^ and *P*‐values from envfit function) of the scores of the five clones on the first and second axis of the PCA (Genetic PC1 and 2 vectors) performed on the IBS distance matrix (Supplementary Fig. S1).

We found an interactive effect between clone and site on the composition of the needle mycobiome and therefore the effect of the clone could not be interpreted alone. We explored whether differences in crown health or diameter were involved in the interaction by selecting sets of clones with the same diameter or crown health between sites and testing if the interaction ‘clone × site’ was still significant after controlling for phenotypic variation. When we selected the five clones with the same diameter in the East–West pair of sites, the interaction ‘clone × site’ was no longer significant (*P* = 0.66) in contrast to when all clones were considered, suggesting that differences in tree diameter at breast height (dbh) across sites was one factor underlying the interaction between clone and site. The same approach could not be taken in the North–South pair of sites because the tree diameters were not overlapping. In the subset of five clones with similar diameter across the East–West sites, both the clone (*P* = 0.008) and the site (*P* = 0.001) had a significant effect on the composition of the mycobiome. Moreover, the composition of the mycobiome was associated with the genetic similarity between the clones, i.e. clones that with higher genetic similarity also displayed more similar mycobiome communities, compared with clones with lower genetic similarity (Fig. 5). The interaction between clone and site was not explained by differences in crown health across sites. When selecting clones with the same crown health across sites, the interaction was still significant in both the North–South pair of sites (*P* = 0.001) and in the East–West (*P* = 0.037).

### Factors explaining alpha and beta diversity of needle mycobiome within sites

We investigated the relative effect of the host genotype, phenotypic traits (dbh and crown health) and location of the tree on the alpha and beta diversity of the needle mycobiome within each of the four sites. For both alpha and beta diversity, the marginal amount of variation explained by the host genotype within each individual site was consistently higher than the variation explained by phenotypic traits, which was always close to zero in most sites and indexes (Supplementary Table S5). Regarding the relative effect of the clone and the spatial distribution of the 10 ramets within sites, we found contrasting patterns for alpha and beta diversity. While in all sites but one, clone explained more variation in beta diversity than the spatial distribution of clones, the opposite was found in alpha diversity where the spatial distribution explained more variation than the clone within all sites (Supplementary Table S5).

## Discussion

The aim of this study was to understand the ecological mechanisms contributing to the assembly of the needle mycobiome of Norway spruce. We showed that host genotype and site were the main factors shaping the composition of the needle mycobiome. The host genotypic effect was associated with the genetic similarity between the Norway spruce clones. The site effect was related to differences in the composition of local air mycobiome. Moreover, the effect of the host genotype on the composition of the mycobiome was site dependent, and it was mainly driven by differences of tree size for the same clone across sites. Altogether, our results suggest that Norway spruce trees recruit their needle mycobiome based on factors comprised of their genotype, the available air mycobiome and the tree size.

### Host genotype influenced the composition of the needle mycobiome across and within sites

The composition of the needle mycobiome differed between Norway spruce clones for both pairs of sites. This host genotype effect was consistently larger than that of the site, and that of the measured phenotypic traits, such as dbh and crown health. We found that the crown health did not influence the diversity of the needle mycobiome. This result is in line with a previous work showing that trees with contrasting physiological state as a result of the infection of a plant pathogen had similar needle mycobiome (Kovalchuk *et al*., 2018). Our results also showed that Norway spruce clones that had higher genetic similarity also had more similar needle mycobiome. The Norway spruce breeding programme, from which our clones were selected, comprises material with different geographical origins (Chen *et al*., 2019). Future studies using more clones than the ones here should explore whether the needle mycobiome is related to traits nested in the demographic origin, as evidenced for other traits in Norway spruce such as tree growth (Milesi *et al*., 2019), needle morphology or is associated with specific genetic variation in the genome (Elfstrand *et al*., 2020).

The genetic control on the mycobiome was found both at community level (PERMANOVA) and when separating the OTUs at different trophic modes and dominant guilds. In both analyses, the amount of variation explained by the genotype was between 8% and 10% for both pairs of sites (*R* ^2^ PERMANOVA = 0.083, average effect size of genotype across guilds = 10.2%). The effect of the genotype reported here is similar to that reported for the phyllosphere microbiome of some perennial (Wagner *et al*., 2016) and annual plants (Sapkota *et al*., 2015), as well as for of other quantitative tree traits such as bud burst, wood density or wood stiffness (Chen *et al*., 2021). We found that the effect of the host genotype was of a similar magnitude across all fungal trophic modes and guilds. This could be because many leaf‐inhabiting fungi have more than one trophic mode and shifts between lifestyles could be triggered by changes in the environmental conditions (Oliva *et al*., 2020a,b). The dominant fungal classes found across all clones in our study coincide with those found in other studies on Norway spruce (Rajala *et al*., 2014) and other *Picea* species (Würth *et al*., 2019).

In our study site explained less variation than the host genotype; however, this has not been the case for previous studies (Eusemann *et al*., 2016). The breadth of the genetic material and environmental conditions are probably behind such inconsistencies. The climatic differences between our pairs of sites are not of the same magnitude as in other studies performed under more contrasting climatic conditions (Eusemann *et al*., 2016; Würth *et al*., 2019). However, the genetic signal in our host population is likely stronger compared to those two studies. First, genome‐wide SNP markers were used to determine the genetic similarity of our clones. Second, the genotypes in our study are derived from most of the natural distribution of Norway spruce, comprising the demographic history of the species (Chen *et al*. 2019).

### Differences in air mycobiome between sites were associated with the needle mycobiome

The composition of the needle mycobiome was associated with changes in air mycobiome across sites and time. We found contrasting air mycobiome communities across sites, which could be the result of the different types of vegetation around the sampled seed orchards or the result of different weather conditions (Castaño *et al*., 2019; Redondo *et al*., 2020). Needle and air communities shared 80% of the OTUs and the presence of certain fungal species in the needles was strongly correlated with its presence in the air mycobiome. This is in line with earlier findings that most of the leaf mycobiome communities are horizontally transmitted (Arnold and Herre, 2003; Arnold, 2007; Rodriguez *et al*., 2009).

Besides the effect of the air mycobiome, we found that differences in the needle mycobiome across sites were also associated with the site‐specific climate. Climate can affect certain tree phenotypic traits, such as needle size or physiology (Rajala *et al*., 2014; Li *et al*., 2018), that in turn determine the composition of fungal communities inhabiting needles; however, climate also affects the distribution of fungal species and therefore the available mycobiome (Castaño *et al*., 2019; Redondo *et al*., 2020).

The composition of the air mycobiome differed between May, when spruce trees elongate their shoots, and July, when shoots are lignifying. The needle mycobiome tended to better reflect the composition of the air mycobiome collected in May and June than in July. One explanation for that is that fresh needles developed during early shoot elongation may be easier to colonize than later in July when the needles have a fully developed cuticle. Alternatively, fungal species sporulating earlier in the season may have a higher chance of establishing and outcompeting late arrivers due to the priority effect (Kennedy *et al*., 2009; Leopold and Busby, 2020).

Although we used different primers for needle and spore traps samples to avoid the amplification of plant DNA in the needle samples, no major biases were expected. The difference between fITS (primers for needles) and gITS (primers spore traps) is that gITS have one more base pair degeneration and amplify some species that fITS do not, e.g. fungi belonging to the genera *Mucor*, *Amanita*, *Cortinarius*, *Melampsora* and *Archaeorhizomyces* (Ihrmark *et al*., 2012). In our spore traps dataset, the OTUs belonging to these genera represent only 0.8% of the total reads.

### Differences in dbh across sites explained the interactive effect between host genotype and site on the needle mycobiome

An interaction between clone and site was identified for beta diversity in both pair of sites. This interaction shows that the relative differences in needle mycobiome between clones varied across sites. As with the effect of the host genotype, this interactive effect was of a similar magnitude across all fungal trophic modes and guilds. Wagner *et al*. (2016) proposed two non‐exclusive mechanisms to understand an interacting effect between host genotype and site: (i) differences in ambient communities, which in our case would refer to the air mycobiome, and (ii) differences in host phenotype between sites. Our results support the effect of both; Norway spruce trees seem to recruit their mycobiome differently across sites depending on their genotype, on the available air mycobiome and on their size. Differences in dbh seemed to be the major driver of the interaction. Nevertheless, although differences in dbh explained the interactive effect between the clone and the site, the effect of dbh within sites was almost negligible. It could be that the differences in dbh within sites were not as large as across sites; therefore, the effect of the tree diameter was overruled by the host genotype effect within each site.

## Conclusions

Tree associated microbes influence host fitness, but what determines the composition of the tree mycobiome is still under investigation, particularly for above ground tissues. In this study, we found that the needle mycobiome was primarily influenced by the tree genotype, and that genetically similar clones displayed a larger similarity in their mycobiomes than clones with less genetic similarity. Besides the genetic effect, Norway spruce trees recruit their fungal counterparts across sites depending on the composition of the air mycobiome available at the site and on the size of the trees. This study provides a foundation for functional analyses aiming to understand whether needle mycobiome may be adaptive to certain environments. These studies will need to use a greater number of host genotypes and will aim at linking host genotypes to specific carbohydrate, amino acid, specialized metabolite or cuticular wax profiles that may determine which microbial taxa can colonize the aerial plant tissues (Li *et al*., 2018; Oliva *et al*., 2020b; Oono *et al*., 2020; Jacoby *et al*., 2021). Some taxa of the plant microbiome are shared between above and below ground plant tissues (David *et al*., 2016; Wagner *et al*., 2016). Future works should explore whether the genetic control that we observed on the needle mycobiome occurs as well in the root fungal communities of Norway spruce, as reported in a pine species (Pérez‐Izquierdo *et al*., 2019).

## Experimental procedures

For sampling, we utilized two pairs of seed orchards containing elite material from the Southern Swedish Norway spruce (*Picea abies* L. Karst) breeding programme (Chen *et al*., 2019) intended for deployment between latitudes 55^°^ and 59^°^ N. The four seed orchards (here ‘sites’) were established in different years with grafted clonal material. Söregärde was planted in 2009, Runesten and Larslund between 2011 and 2012, and Gåtebo in 2012. The establishment of the sites was also done in a pairwise design (Fig. 1), so that Norway spruce clones are only shared between each pair of sites. None of the clones in this study are present in all sites (Supplementary Table S1).

We selected 15 Norway spruce genotypes (‘clones’) shared between the two sites in the North–South pair (Larslund and Söregärde), and another 15 Norway spruce clones shared in the East–West pair of sites (Gåtebo and Runesten). From each of these clones, we sampled 10 vegetatively propagated (Rosvall, 2019) trees (‘ramet’), i.e. a total of 150 trees/seed orchard (Fig. 1A and B). The Norway spruce clones were selected based on their spring phenology (flushing category) in combination with their frequency at the pair of sites. The spring phenology for each clone was scored in May/June 2015 according to the Krutzsch scale (Krutzsch, 1973) and thereafter classified as flushing ‘early’, ‘medium’ or ‘late’ relative to the site average.

### Genetic similarity between Norway spruce clones

The genetic similarity between the Norway spruce tree clones of this study was investigated by collecting the unfiltered SNP variant data from an existing genotyping data set (Vidalis *et al*., 2018; Baison *et al*., 2019; Bernhardsson *et al*., 2019). Variants were filtered according to Bernhardsson *et al*. (2019) with a few modifications. Briefly, biallelic SNPs within the extended probe regions were filtered with QualbyDepth > 2.0, FisherStrand < 60.0, RMSMappingQuality (MQ) > 40, MappingQualityRankSumTest (MQRankSum) > −12.5, ReadPosRankSumTest (ReadPosRankSum) > −8.0, StrandOddsRatio (SOR) < 3.0 using VCFtools (Danecek *et al*., 2011). Variants called in possible collapsed regions of the assembly were removed by filtering for depth between 6 and 40, GQ < 15, mean depth between 10 and 30, 20% missing data and minor allele count 1. Additionally, only variants with a *P*‐value of excess of heterozygosity higher than 1 e‐10 were kept avoiding collapsed reads. To obtain the genetic similarity between tree clones according to the 160 992 filtered SNPs, we did a principal component analysis (PCA) based on an identity‐by‐state matrix (IBS) using PLINK 1.9 (Chang *et al*., 2015) (Supplementary Fig. S1). Based on the IBS matrix, a dendrogram was also obtained using hierarchical clustering in R.

### Sampling of Norway spruce needles

The needle sampling was performed in August 2017. At each site, 10 shoots per ramet were randomly picked from the crown in all directions ranging from the lowest branches of the tree to 2 m height, pooled and kept in a paper bag for 3–5 days until future processed. At sampling, the dbh, and crown health status were recorded for each of the sampled tree (Supplementary Table S1). The crown health was evaluated in a scale from 0 to 5, in which 0 = dead tree, 1 = tree unlikely to recover from widespread stress symptoms, 2 = tree with discoloration of needles/needle loss in most of the crown, 3 = tree with some signs of stress, flagging of individual branches and/or localized fungal or insect attacks, 4 = tree with no sign of stress, no signs of needle loss or ongoing fungal/insect/herbivore attacks, and 5 = tree with no sign of stress, nor previous fungal/insect/herbivore attacks. The current‐year needles, i.e. the needles of the fresh sprout of the collecting year, of all the 10 shoots per ramet were manually excised from the shoot and surface sterilized following the protocol of Arnold *et al*. (2007) consisting of sequential immersions in 95% EtOH for 5 s, 0.5% NaCl solution for 2 min, 70% EtOH for 2 min and thereafter rinsed with sterile water for 5 min. Needles were then freeze dried, homogenized with a ball mill, and kept at −20°C until DNA extraction.

### Spore trap collections for air mycobiome characterization

At each site, 10 passive spore traps were placed in a grid pattern across the site. The passive spore traps consisted of a piece of filter paper (Whatmann no. 1; 90‐mm‐diameter, GE Healthcare, Chicago, IL, USA), previously soaked in 4× TE buffer (Schweigkofler *et al*., 2004). Each filter paper was then placed on a metal rack at 1.5 m height, as described by Redondo *et al*. (2020). Samples were collected weekly from May to July 2017, except at Gåtebo, where samples were collected only in June and July. Filters were stored at −20°C until DNA extraction. To elute the spores for DNA extraction, we used a modified version of the washing described by Schweigkofler *et al*. (2004). The modification was that we used 20 ml of a different buffer [50 mM Tris (pH 8), 50 mM disodium ethylenediaminetetraacetate (EDTA), 3% sodium dodecyl sulfate and 1 M NaCl]. After the last washing step, 20 ml of 100% isopropanol was added to the suspension and centrifuged to concentrate the spores. In total, 322 spore trap samples were collected. Before DNA extraction, paper filters were washed following the steps described by Redondo *et al*. (2020).

### DNA extraction, library preparation and amplicon sequencing

DNA from needles was extracted using the CTAB protocol following Ihrmark *et al*. (2012), whereas DNA from spore traps was obtained with the NucleoSpin® Soil kit (Macherey‐Nagel, Hoerdt, France). Libraries for needles and spore traps were prepared separately. For needle samples, we amplified the fungal ITS2 region by using the fITS7 (Ihrmark *et al*., 2012) and ITS4 (White *et al*., 1990) primers. We used the fITS7 primers because of its higher specificity towards fungal DNA and to reduce the amplification of plant DNA. For the spore traps, ITS2 region was amplified using the gITS7 (Ihrmark *et al*., 2012) and ITS4 (White *et al*., 1990) primers. For both needle and spore trap samples, primers were tagged with 8‐basepair tags constructed with Barcrawl software (Frank 2009). For the PCR reaction, we used three technical replicates from each of the ramets (biological replicates) as well as for each of the spore traps collected at a specific time point. The number of PCR cycles were optimized for each sample to establish the minimum number of cycles at which the PCR product was visible in an agarose gel avoiding the saturation phase of the PCR reaction (Clemmensen *et al*., 2016). For both needles and spore traps, the optimum number of cycles ranged between 25 and 34 cycles. After quantifying the amount of pcr‐product DNA with a Qubit fluorometer, equal mass per sample were pooled prior to sequencing. The pooled samples were purified with the AMPure kit (Beckman Coulter, Brea, CA, USA). Adaptor ligation and Pacific Biosciences (PacBio) amplicon sequencing was performed at SciLifeLab facilities (NGI, Uppsala). Needle and spore trap amplicons were sequenced separately using the PacBio Sequel, and SMRT RSII technology, respectively.

### Sequence analysis and taxonomic classification

Sequences were filtered for quality, clustered and de‐multiplexed using the SCATA pipeline (http://scata.mykopat.slu.se). Sequences with an average quality score < 10, base average quality < 2, lacking the primer sites (primer match value = 0.9) or containing false combinations of used primer tags, i.e. tag jumps (Schnell *et al*., 2015) were removed. A total of 2 745 030 raw reads were obtained, from which 1 012 231 and 821 184 passed quality control for spore trap and needle communities, respectively. The average read count for needle and spore samples was 1806 and 279, respectively. The average read length was 267 bp for needle samples and 261 bp for spore samples. Sequences were clustered in operational taxonomic units (OTUs) using a threshold distance of 1.5% and a penalty of 1 for mismatch. OTUs with less than 10 reads, or that appeared only in one sample were removed from the dataset. Samples with less reads than the 10% quantile of average reads per sample (250 for needles and 150 for spore traps) were also discarded. A summary of samples included in the analysis is available in Supplementary Table S1. Each of the OTUs was taxonomically assigned using Protax software (Somervuo *et al*., 2016; Abarenkov *et al*., 2018) implemented in PlutoF (https://plutof.ut.ee) using a threshold value of 0.5 (plausible classification) (Abarenkov *et al*., 2018). By using a taxonomic classification database (index fungorum) and a reference sequence database (Unite), PROTAX provides a statistically calibrated probability for the taxonomical placement of the ITS sequence across six taxonomical levels from phylum to species as well as the reference sequence with the best match with the query sequence. PROTAX accounts for gaps in the taxonomy (incertae sedis) database by creating dummy nodes that are different from those reads that cannot be assigned at a taxonomical level and are classified as unknown. To exclude OTUs belonging to plants, we performed a least common ancestor analysis with the software MEGAN (Huson *et al*., 2007), with a min‐score of 300 and a minimum identity of 90%, on the OTUs that could not be assigned at any phylum level by Protax. The OTUs classified as ‘Fungi’ in MEGAN were retained as ‘Fungi, unknown phylum’ and merged with the OTUs previously classified as fungi by Protax. The trophic mode and guild of each OTU was then obtained by using FUNguild (Nguyen *et al*., 2016).

### Analysis of the needle mycobiome across sites

The relative contribution of the host genotype, site, their interaction, and tree phenotypic traits (dbh, crown health and flushing category) was tested on the alpha and beta diversity of the needle mycobiome across sites. The analyses were done separately for the two pairs of sites. To control for the fact that trees had different sizes between sites, as a result of being planted at different years, the diameter values were standardized for each clone within sites prior to the analyses. For all diversity analyses a non‐rarefied OTU table was used, and the square root of the total number of reads per sample as the first factor in all models was included. By doing this, we explicitly accounted for the potential bias resulting from different sequencing depth instead of rarefying (McMurdie and Holmes, 2014; Bálint *et al*., 2015; Wagner *et al*., 2016).

For alpha diversity calculations, the OTU richness and Shannon index were obtained using the vegan package (Oksanen *et al*., 2019), and their association with read counts, clone, site, clone × site and phenotypic traits were tested using linear regressions. For beta diversity, a Hellinger transformed OTU table was used. A PERMANOVA was performed by using the adonis2 function in the vegan package in the R environment to test the significance of the two main effects ‘clone’, ‘site’, their interaction, the effect of phenotypic traits and total read counts. Since the flushing category depend on the genotype (i.e. clone), both factors could not be included in the models together. The analysis was therefore repeated using flushing category instead of the clone, to determine which one explained the most variation. The differences in community composition were visualized using principal coordinate analysis using Bray–Curtis distance and implemented with the Phyloseq package (McMurdie and Holmes, 2013). We calculated the amount of variation in relative abundance of each OTU that was explained by clone, site and their interaction. A logistic regression was built for each OTU and we used the values from the ANOVA table to obtain the adjusted proportion of the sums of squares for each factor (*ω* ^2^), i.e. effect size, following the formula *ω* ^2^ = (SSQ_effect_ − df_effect_ × MS_error_)/(SSQ_total_ + MS_error_) found on http://onlinestatbook.com, where SSQ is the sum of squares, df is the degrees of freedom and MS is the mean squares. The *ω* ^2^ values were grouped by the effect size by trophic modes and most dominant guilds. To account for the fact that some OTUs belonged to more than one guild, the guilds and trophic modes were converted to dummy variables and assigned as 1 (presence) or 0 (absence) values of each guild and trophic mode to each OTU (Redondo *et al*., 2020).

To test whether differences in crown health or dbh were causing the interaction clone × site, a set of clones with the same diameter or crown health status between sites were selected and tested if the interaction clone × site was still significant. We found that when considering clones with the same diameter between sites (*n* = 5) of the East–West pair, the effect of ‘clone × site’ interaction was no longer significant. Only on those five clones in East–West pair of sites were further tested whether their genetic similarity was associated with the composition of their mycobiome. For this, the scores of each clone on the first and second axis of the PCA displaying their genetic similarity were obtained and fitted on the ordinations of the needle mycobiome using the envfit function in the vegan package.

To test to which extent differences in climate and weather across sites influenced the composition of the needle mycobiome, we collected values of mean yearly air temperature and total yearly precipitation of the last 20 years (August 1997 to August 2017, i.e. climate) and also of the year before the sampling (August 2016 to August 2017, i.e. weather) from the closest active climatic station to each of the sites (www.smhi.se). These data were included as covariables in a global analysis of the composition of the needle mycobiome across all four sites. PERMANOVA analysis was performed for climate and weather separately.

### Analysis of the air mycobiome and its association with the needle microbiome across sites

The composition of the air mycobiome was compared for each pair of sites for the accumulated inoculum on each spore traps, i.e., pooling all the sequencing data from each week for each spore trap within each site, and also separating these samples weekly and monthly. We tested the association between the differences in air mycobiome on the composition of needle communities on each of the pair of sites. For this, we tested the correlation of the fold change differences in air and needle mycobiome across the two targeted sites for all OTUs with a linear regression. This would show whether increasing/decreasing relative abundance of an OTU in the air mycobiome is associated with its increase/decrease relative abundance in the needle mycobiome across sites. To test how the composition of the air mycobiome affected the composition of the needle mycobiome in the whole dataset, we used the centroid values of the pooled spore trap samples of each four sites as a covariables in a global analysis of the composition of the needle mycobiome across all four sites.

The air mycobiome was subdivided by month for May, June and July to identify which month had the highest similarity to the needle mycobiome. A principal coordinate analysis was performed for each site including needle samples and spore trap samples. The similarity between needle and spore trap communities was obtained for each month and site based on the *R* ^2^ value of a PERMANOVA (adonis2 function in vegan package) using the variable ‘origin’, i.e., either needles or spore traps, as explanatory. This analysis was performed using both Bray–Curtis and Jaccard index.

### Analysis of the needle mycobiome within sites

Within each site, the contribution of host genotype, phenotype and relative location of the tree in the site (spatial) was tested based on alpha and beta diversity. The spatial descriptors, i.e. the distance‐based Moran eigenvector maps (dbMEM), were obtained using the adespatial package in the R environment (Dray *et al*., 2020). Within each site, the trees were equally spaced and the number of row and column in which each tree was located within each site was used to obtain the spatial descriptors. A forward selection on the dbMEM was performed to test how much of the variation in alpha and beta diversity was explained by these spatial descriptors. For alpha diversity (OTU richness and Shannon index), the step function was used to select the model with the lowest AIC value. For OTU richness, we included 9, 6, 2 and 8 dbMEM for Larslund, Söregärde, Runesten and Gåtebo, respectively. For Shannon index, we had 5, 10, 4 and 4 dbMEM for Larslund, Söregärde, Runesten and Gåtebo, respectively. For beta diversity, a forward selection of the dbMEM was performed with the forward.sel function of the adespatial package. We included 3, 2, 2 and 2 dbMEM for Larslund, Söregärde, Runesten and Gåtebo, respectively. The selected dbMEM, together with clone and phenotypic traits (dbh and crown health) were included on a variation partitioning analysis in which the varpart function of the vegan package (Oksanen *et al*., 2019) was used.

## Supporting information


Appendix S1: Supplementary Information

Appendix S2: Table S2

## Data Availability

The datasets of this publication, including the OTU tables, metadata, taxonomical classification, consensus sequence of each OTU and the IBS matrix of the tree genotypes are archived in Figshare: https://doi.org/10.6084/m9.figshare.13663586.v3. The raw sequencing files together with the tag files for demultiplexing and clustering in SCATA (http://scata.mykopat.slu.se) are also available in Figshare: https://doi.org/10.6084/m9.figshare.17113361.v3.
