# Supplementary material for: Host genotype interacts with aerial spore communities and influences the needle mycobiome of Norway spruce
Source: Environ Microbiol. 2022 Mar 22;24(8):3640–54. doi: 10.1111/1462-2920.15974 (PMC9544151; doi:10.1111/1462-2920.15974)
Supplement: Supplementary file 1 — Appendix S1: Supplementary Information [file EMI-24-3640-s001.docx]

**Supplementary Table S1**. Norway spruce clones with their number of samples that amplified the ITS region during library preparation (n), the genetic origin based on (Chen et al., 2019) and their phenotypic information. Diameter at breast height (dbh) was measured in cm.

|  |  |  | East-West pair of sites | | | | | | |  | North-South pair of sites | | | | | | |
| --- | --- | --- | --- | --- | --- | --- | --- | --- | --- | --- | --- | --- | --- | --- | --- | --- | --- |
|  |  |  | Gåtebo | | |  | Runesten | | |  | Larslund | | |  | Söregärde | | |
| Clone | Flushing category | Genetic origin | n | dbh | crown health |  | n | dbh | crown health |  | n | dbh | crown health |  | n | dbh | crown health |
| 168 | Late | Central Sweden | 8 | 23.8 | 4.9 |  | 5 | 45.6 | 4.6 |  |  |  |  |  |  |  |  |
| 206* | Early | Alpine | 5 | 22.0 | 5.0 |  | 1 | 27.0 | 5.0 |  |  |  |  |  |  |  |  |
| 222 | Medium | Alpine | 4 | 15.0 | 4.8 |  | 8 | 31.4 | 4.4 |  |  |  |  |  |  |  |  |
| 232 | Medium | Northern Poland | 5 | 28.4 | 4.8 |  | 7 | 36.7 | 3.7 |  |  |  |  |  |  |  |  |
| 239 | Late | Alpine | 5 | 29.8 | 4.6 |  | 6 | 32.2 | 4.3 |  |  |  |  |  |  |  |  |
| 323 | Early | Alpine | 6 | 22.3 | 4.8 |  | 5 | 48.2 | 4.6 |  |  |  |  |  |  |  |  |
| 372 | Early | Alpine | 8 | 24.9 | 4.9 |  | 5 | 37.4 | 3.6 |  |  |  |  |  |  |  |  |
| 507 | Medium | Central Europe | 3 | 18.3 | 4.7 |  | 4 | 48.0 | 4.0 |  |  |  |  |  |  |  |  |
| 714 | Late | Romania | 7 | 25.7 | 4.7 |  | 8 | 33.1 | 4.1 |  |  |  |  |  |  |  |  |
| 789 | Late | Central Europe | 9 | 20.2 | 4.8 |  | 7 | 34.3 | 3.7 |  |  |  |  |  |  |  |  |
| 798 | Early | Alpine | 5 | 14.6 | 4.8 |  | 5 | 28.6 | 3.4 |  |  |  |  |  |  |  |  |
| 903 | Early | Alpine | 7 | 23.3 | 4.3 |  | 10 | 55.2 | 4.5 |  |  |  |  |  |  |  |  |
| 946 | Late | Russian-Baltic | 6 | 22.7 | 5.0 |  | 10 | 27.4 | 3.4 |  |  |  |  |  |  |  |  |
| 1071 | Late | Central Europe | 5 | 24.0 | 4.8 |  | 9 | 44.4 | 3.9 |  |  |  |  |  |  |  |  |
| 7455 | Late | Romania | 8 | 16.1 | 4.5 |  | 9 | 38.3 | 4.4 |  |  |  |  |  |  |  |  |
| 329 | Early | Alpine |  |  |  |  |  |  |  |  | 9 | 54.3 | 4.8 |  | 8 | 122.4 | 4.8 |
| 402 | Early | Alpine |  |  |  |  |  |  |  |  | 9 | 51.3 | 4.6 |  | 10 | 104.2 | 4.3 |
| 405 | Early | Alpine |  |  |  |  |  |  |  |  | 9 | 41.4 | 4.7 |  | 7 | 83.6 | 5.0 |
| 443 | Early | Central Europe |  |  |  |  |  |  |  |  | 8 | 46.4 | 4.9 |  | 9 | 109.9 | 4.8 |
| 1069 | Early | Russian-Baltic |  |  |  |  |  |  |  |  | 7 | 58.0 | 5.0 |  | 8 | 98.5 | 4.9 |
| 1137 | Medium | Russian-Baltic |  |  |  |  |  |  |  |  | 6 | 45.7 | 4.8 |  | 7 | 81.3 | 4.9 |
| 1221 | Late | Romania |  |  |  |  |  |  |  |  | 9 | 64.4 | 5.0 |  | 7 | 114.0 | 5.0 |
| 1245 | Late | Russian-Baltic |  |  |  |  |  |  |  |  | 9 | 51.6 | 5.0 |  | 10 | 128.7 | 4.5 |
| 1560 | Medium | Romania |  |  |  |  |  |  |  |  | 7 | 61.9 | 4.9 |  | 10 | 121.9 | 4.9 |
| 2009 | Late | Romania |  |  |  |  |  |  |  |  | 10 | 53.5 | 5.0 |  | 7 | 107.8 | 4.3 |
| 3087 | Medium | Central Europe |  |  |  |  |  |  |  |  | 9 | 51.7 | 4.9 |  | 7 | 98.3 | 4.7 |
| 3812 | Medium | Romania |  |  |  |  |  |  |  |  | 9 | 54.9 | 4.8 |  | 9 | 110.6 | 4.3 |
| 6669 | Medium | Central Europe |  |  |  |  |  |  |  |  | 10 | 57.5 | 4.9 |  | 10 | 112.7 | 4.9 |
| 7300 | Late | Romania |  |  |  |  |  |  |  |  | 10 | 57.6 | 5.0 |  | 7 | 119.0 | 4.9 |
| 8280* | Medium | Alpine |  |  |  |  |  |  |  |  | 2 | 64.0 | 4.8 |  | 8 | 106.7 | 5.0 |

***** Clones 206 and 8220 were not included in the analysis because less than 3 replicates were present in one location after library preparation.

**Supplementary Table S2**. Relative proportions of each of the fungal classes averaged for each of the Norway spruce clones included in the study. See separated Supplementary_table_S2.xlsx file.

**Supplementary Table S3**. Taxonomical classification of the 20 most dominant OTUs and their relative proportion on the overall dataset.

| OTU name | Proportion  of total reads | Phylum | Class | Order | Family | Genus | Species |
| --- | --- | --- | --- | --- | --- | --- | --- |
| scata4352_0 | 37% | Ascomycota | Dothideomycetes | Capnodiales | Cladosporiaceae | *Cladosporium* | unknown |
| scata4352_2 | 13% | Ascomycota | Dothideomycetes | Dothideales | Dothioraceae | *Aureobasidium* | *Aureobasidium pullulans* |
| scata4352_4 | 9% | Ascomycota | Dothideomycetes | Dothideales | dummy_family_Celosporium | *Celosporium* | unknown |
| scata4352_3 | 7% | Ascomycota | Dothideomycetes | Dothideales | Dothioraceae | *Sydowia* | *Sydowia polyspora* |
| scata4352_10 | 3% | Ascomycota | Sordariomycetes | Sordariales | Sordariaceae | *Sordaria* | unknown |
| scata4352_8 | 3% | Ascomycota | Leotiomycetes | Helotiales | Sclerotiniaceae | *Botrytis* | unknown |
| scata4352_6 | 2% | Ascomycota | Dothideomycetes | Pleosporales | Didymellaceae | *Epicoccum* | unknown |
| scata4352_7 | 2% | Ascomycota | Dothideomycetes | Pleosporales | Pleosporaceae | *Alternaria* | unknown |
| scata4352_5 | 2% | Basidiomycota | Tremellomycetes | Tremellales | Bulleribasidiaceae | *Vishniacozyma* | unknown |
| scata4352_16 | 2% | Ascomycota | Dothideomycetes | Pleosporales | Pleosporaceae | *Alternaria* | unknown |
| scata4352_12 | 2% | Basidiomycota | Microbotryomycetes | Sporidiobolales | dummy_family_Sporobolomyces | *Sporobolomyces* | unknown |
| scata4352_11 | 1% | Ascomycota | Sordariomycetes | Xylariales | dummy_family_Tristratiperidium | *Tristratiperidium* | unknown |
| scata4352_9 | 1% | Basidiomycota | Pucciniomycetes | Pucciniales | Coleosporiaceae | *Chrysomyxa* | unknown |
| scata4352_15 | 1% | Ascomycota | Dothideomycetes | Pleosporales | Didymellaceae | *Ascochyta* | unknown |
| scata4352_14 | 1% | Basidiomycota | Tremellomycetes | Filobasidiales | Filobasidiaceae | *Filobasidium* | unknown |
| scata4352_19 | 1% | Ascomycota | Dothideomycetes | Pleosporales | Sporormiaceae | *Preussia* | unknown |
| scata4352_17 | 1% | Ascomycota | Dothideomycetes | Pleosporales | Sporormiaceae | *Preussia* | unknown |
| scata4352_13 | 1% | Ascomycota | Dothideomycetes | Pleosporales | Phaeosphaeriaceae | *Phaeosphaeria* | unknown |
| scata4352_18 | 1% | Ascomycota | Dothideomycetes | Pleosporales | Sporormiaceae | *Sporormiella* | *Sporormiella intermedia* |
| scata4352_23 | 1% | Ascomycota | Dothideomycetes | Pleosporales | Montagnulaceae | *Paraphaeosphaeria* | unknown |

**Supplementary Table S4**. Output of the PERMANOVA analysis including the climate, weather and air mycobiome composition. The analysis was performed on all four sites. We could not include all climatic variables, weather variables and centroids in a single PERMANOVA because we have only four sites and the model did not fit owing to collinearity between the covariables

|  | Factor | df | R^2^ | *P* |
| --- | --- | --- | --- | --- |
| Model including climatic variables (20 years) |  |  |  |  |
|  | Read count | 1 | 0.017 | **0.0001** |
|  | Precipitation (20 years) | 1 | 0.042 | **0.0001** |
|  | Temperature (20 years) | 1 | 0.046 | **0.0001** |
|  | Site | 1 | 0.020 | **0.0001** |
|  | Clone ID | 26 | 0.077 | **0.0001** |
|  | Diameter (DBH) | 1 | 0.0027 | 0.14 |
|  | Crown health | 1 | 0.0029 | 0.08 |
|  | Residuals | 388 | 0.79 | **-** |
| Model including weather variables (year before the sampling) |  |  |  |  |
|  | Read count | 1 | 0.017 | **0.0001** |
|  | Precipitation (1 year) | 1 | 0.037 | **0.0001** |
|  | Temperature (1 year) | 1 | 0.036 | **0.0001** |
|  | Site | 1 | 0.036 | **0.0001** |
|  | Clone ID | 26 | 0.077 | **0.0001** |
|  | Diameter (DBH) | 1 | 0.0027 | 0.11 |
|  | Crown health | 1 | 0.003 | 0.10 |
|  | Residuals | 388 | 0.79 | - |
| Model including the centroid values of the air mycobiome |  |  |  |  |
|  | Read count | 1 | 0.017 | **0.0001** |
|  | Centroid in Axis 1 | 1 | 0.047 | **0.0001** |
|  | Centroid in Axis 2 | 1 | 0.035 | **0.0001** |
|  | Site | 1 | 0.027 | **0.0001** |
|  | Clone ID | 26 | 0.077 | **0.0001** |
|  | Diameter (DBH) | 1 | 0.0028 | 0.12 |
|  | Crown health | 1 | 0.0029 | 0.085 |
|  | Residuals | 388 | 0.79 | **-** |

**Supplementary Table S5**. Summary of the variation partitioning for alpha and beta diversity. Analyses were performed on each site separately. Non-shared effects represent the marginal fractions of the variation explained by each factor. Shared effects are the intersection value between each pair of factors and the intersection of all three.

|  | Alpha diversity | | | | | | | | |  | Beta diversity | | | | |
| --- | --- | --- | --- | --- | --- | --- | --- | --- | --- | --- | --- | --- | --- | --- | --- |
|  | North-South | | | |  | East-West | | | |  | North-South | |  | East-West | |
|  | Larslund | | Söregärde | |  | Runesten | | Gåtebo | |  | Larslund | Söregärde |  | Runesten | Gåtebo |
|  | OTU  richness | Shannon  index | OTU  richness | Shannon  index |  | OTU  richness | Shannon  index | OTU  richness | Shannon  index |  |  |  |  |  |  |
| *Non-shared effects* |  |  |  |  |  |  |  |  |  |  |  |  |  |  |  |
| Clone | 0% | 1.8% | 2.8% | 9.3% |  | 1.7% | 3.6% | 3.5% | 0% |  | 6.8% | 3.7% |  | 2% | 0.4% |
| Spatial* | 7.7% | 9.7% | 12.2% | 11.8% |  | 2.8% | 8.5% | 13.8% | 10.8% |  | 2% | 1.3% |  | 1.8% | 1.7% |
| Phenotype** | 0% | 0% | 3.2% | 0.3% |  | 0.8% | 3.3% | 0% | 0% |  | 0% | 0.3% |  | 0% | 0% |
| *Shared effects* |  |  |  |  |  |  |  |  |  |  |  |  |  |  |  |
| Clone ∩ Spatial | 3.4% | 3.5% | 3.3% | 2.3% |  | 0% | 0.9% | 0.3% | 0% |  | 1% | 0.4% |  | 1.3% | 0% |
| Clone ∩ Phenotype** | 0.5% | 0.1% | 0% | 0.9% |  | 0.1% | 0% | 0% | 0.6% |  | 0.1% | 0% |  | 0.3% | 0.1% |
| Spatial ∩ Phenotype** | 0.1% | 0% | 1.7% | 1.3% |  | 0% | 2% | 1.3% | 0% |  | 0.1% | 0.2% |  | 0% | 0% |
| Clone ∩ Spatial ∩ Phenotype** | 0.1% | 0.2% | 0% | 0% |  | 0.7% | 0.3 | 0% | 0% |  | 0% | 0% |  | 0% | 0.1% |

* Spatial refers to the spatial descriptors obtained from the relative position of the tree within the seed orchard.

**The factor phenotype included both the diameter at breast height and the crown health.

**Supplementary Figure S1.** Plot of the Principal component analysis of the genetic variation of the Norway spruce clones included in this study. The five clones labeled are the ones used in the analyses that correlates their genetic distance and the composition of their mycobiome (Figure 5 in main text).

**Supplementary Figure S2.** Ordination plots of principal coordinate analysis of (a) north-south and (b) east-west pair of sites. The main circles and bars represent the centroid and standard error of the samples from each of the clones. The background circles represent each of the samples. Ordination plots are faceted by clone, so that the relative coordinates of each centroid are comparable across all clones for each of the two panels.

**Supplementary Figure S3**. Comparison of weekly-based air mycobiome composition across sites for the North-South pair of sites (a) and for the East-West pair of sites (b). Plots depict principal coordinate analysis separated by sites. The main circles and bars represent the centroid and standard error of the samples from each site. The small circles represent each of the weekly collected samples. The R^2^ and *P* values are based on a PERMANOVA analysis performed with the adonis2 function in R.

**Supplementary Figure S4.** Comparison between needle and air mycobiome over the three months where spore traps were collected for the (a) North-South, and (b) East-West pair of sites. Each of the rows of the panels corresponds to one of the three months where traps were collected. Top row=May, middle row=June, bottom row=July. Similarities in community composition between needles (circles) and spore traps (squares) for each of the three months (May to July) for the north-south and for the east-west pair of sites are displayed by the R^2^ value of a PERMANOVA in which the source of the communities (spore traps or needles) were included as a factor. We display the R^2^ values of the PERMANOVA using both Bray-curtis and Jaccard indexes. Lower R^2^ values indicate more similarity between spore traps and needle communities.
